# Impact of acquisition and reconstruction parameters on quantitative accuracy in dual‐layer spectral CT: A phantom study

**DOI:** 10.1002/acm2.70423

**Published:** 2025-12-15

**Authors:** Lingxuan Leng, Qizhen Zhu, HuiYing Qu, Bing Zhou, Wenbo Li, Fuquan Zhang, Bo Yang, Jie Qiu

**Affiliations:** ^1^ Department of Radiation Oncology Peking Union Medical College Hospital Chinese Academy of Medical Sciences and Peking Union Medical College Beijing China; ^2^ Department of Radiation Oncology State Key Laboratory of Complex Severe and Rare Diseases Peking Union Medical College Hospital Chinese Academy of Medical Science and Peking Union Medical College Beijing China

**Keywords:** effective atomic number, radiation therapy planning, relative electron density, spectral CT, virtual monochromatic images

## Abstract

**Purpose:**

This study aims to systematically evaluate the impact of different CT acquisition and reconstruction parameters on the accuracy of dual‐layer spectral CT‐based functional imaging, including effective atomic number (Z_eff_) maps, relative electron density (RED) maps, and virtual monochromatic images (VMIs).

**Methods:**

A standardized phantom equipped with various material inserts, including air, soft‐tissue–equivalent, polymer, and bone‐equivalent materials, was scanned using a dual‐layer spectral CT system. The investigated acquisition and reconstruction parameters included tube voltage, tube current–time product (mAs), reconstruction thickness, pitch, and reconstruction filter. Quantitative accuracy was assessed for RED and Z_eff_ by comparison with manufacturer‐provided reference values, using Bland–Altman analysis. Comparative analyses were performed between VMIs at 40, 70, and 100 keV and conventional CT (120 kVp single‐energy reconstruction from the dual‐layer system) by calculating the mean and standard deviation of Hounsfield units (HU) differences, to evaluate the dependence of CT number stability on acquisition and reconstruction parameters.

**Results:**

Across all acquisition and reconstruction parameters, RED and Z_eff_ measurements remained accurate, with signed differences within 2.4% and 0.4%, respectively. Compared with conventional CT, VMIs demonstrated greater robustness to parameter change. At 70 keV, HU variations did not exceed 8 HU, whereas conventional CT exhibited deviations up to 118 HU. Low‐energy VMIs (40 keV) showed greater variability, with a maximum difference of 27 HU. Among the investigated parameters, tube voltage exerted the most evident influence.

**Conclusion:**

Dual‐layer spectral CT demonstrated quantitative accuracy in functional imaging (ED, Z_eff_, and VMIs) across diverse acquisition and reconstruction parameters.

## 1 INTRODUCTION

Computed tomography (CT) simulation plays a critical role in radiation therapy. Anatomical information from CT scans is used to delineate target volumes and organs at risk (OARs), and attenuation data are used to calculate doses.^1^ Radiotherapy treatment planning relies on converting CT Hounsfield units (HU) to relative electron density (RED) or stopping power ratio (SPR) through calibration curves.^2^, ^3^ However, single‐energy CT (SECT) has limitations in this conversion process. SECT measures integrated radiation attenuation across the entire x‐ray spectrum, suffers from beam hardening effects, and cannot distinguish between materials with similar attenuation but different elemental compositions.^4^ These uncertainties can result in dose delivery inaccuracies, particularly in proton therapy, where SPR errors directly contribute to range uncertainty.^5^ Moreover, calibration curves require fixed scan protocols, thereby limiting patient‐specific image optimization. Spectral CT overcomes the limitations of SECT by acquiring multi‐energy attenuation data to generate additional spectral images beyond SECT data, including virtual monochromatic images (VMIs), RED maps, effective atomic number (Z_eff_) maps, and iodine concentration maps.^6^ Crucially, spectral CT enables direct quantification of RED and Z_eff_ for dose calculation without relying on calibration curves. Evaluating the quantitative accuracy of RED and Z_eff_ measurements under varying acquisition and reconstruction parameters is a critical step toward the implementation of spectral CT‐based dose calculation in radiotherapy planning.^7^

Spectral CT enables the reconstruction of VMIs that simulate imaging acquisition at specific photon energy levels. Low‐energy VMIs (40–70 keV) have been shown to enhance lesion conspicuity and CNR in abdominal and head‐and‐neck malignancies, enabling more accurate identification and segmentation of tumors and adjacent normal tissues.^8^, ^9^ In contrast, high‐energy VMIs (≥100 keV) are effective in reducing artifacts caused by metallic implants, which can otherwise compromise both anatomical contouring and dose calculation accuracy.^10^ Despite these promising results, the quantitative reliability and robustness of VMI reconstructions under varying acquisition and reconstruction parameters remain insufficiently characterized. Therefore, comprehensive validation of VMI accuracy across different acquisition settings is essential before spectral CT‐derived VMIs can be fully integrated into clinical radiotherapy workflows.

Spectral CT can be implemented through several technological approaches, including dual‐source, rapid kVp‐switching, and dual‐layer detector systems. Previous studies have demonstrated the feasibility of RED, Z_eff_, and monochromatic CT number quantification using twin‐beam, dual‐source, and kVp‐switching spectral CT.^11^, ^12^ Although investigations on dual‐layer spectral CT have also been reported, most remain limited to specific patient cohorts and imaging modalities, lacking comprehensive evaluation of the synergistic effects of multiple acquisition and reconstruction parameters.^13^, ^14^, ^15^ Furthermore, existing studies frequently fail to account for the variability in multi‐parameter imaging and scan protocols when assessing the robustness of spectral CT performance. Therefore, a critical research gap persists regarding the comprehensive evaluation of the stability of dual‐layer spectral CT across diverse acquisition and reconstruction parameters.

In this study, we focused on five key acquisition and reconstruction parameters: tube voltage, tube current–time product (mAs), slice thickness, reconstruction filter, and pitch, which are most frequently adjusted in clinical CT protocols and directly influence both image quality and quantitative accuracy. Tube voltage and mAs define the x‐ray spectrum and photon statistics, thereby affecting spectral separation and noise. In pediatric imaging, for example, tube voltage is often reduced to achieve lower radiation dose.^14^ Slice thickness and reconstruction filter determine the balance between spatial resolution and noise, influencing the stability of quantitative measurements.^16^ Pitch governs the sampling geometry in helical acquisitions. Previous phantom studies in fast kVp‐switching dual‐energy CT have shown that increasing pitch leads to higher image noise, reduced spatial resolution, and decreased stability of iodine quantification.^17^

This study aims to systematically evaluate the accuracy of spectral CT‐derived functional imaging, including RED, Z_eff_, and VMIs, under varying acquisition and reconstruction parameters using a standardized phantom. The evaluation specifically focuses on the quantitative accuracy of RED and Zeff, and the energy‐level stability of CT numbers in VMIs. By assessing the influence of key acquisition and reconstruction parameters, we intend to determine the reliability and robustness of dual‐layer spectral CT for clinical applications in radiation therapy. Our findings will offer critical insights into the quantitative stability of dual‐layer spectral CT and provide an empirical foundation for clinical protocol optimization, supporting its integration into precision radiotherapy planning and functional tissue characterization.

## 2 MATERIALS AND METHODS

### 2.1 CT scanner and phantom

The present study utilized the Spectral CT 7500 scanner, a dual‐layer detector‐based spectral CT system. The upper detector layer incorporates a low‐density yttrium‐based scintillator (yttrium gadolinium oxide, YGO) optimized for low‐energy photon absorption, while the lower layer contains a high‐density gadolinium oxysulfide (GOS) scintillator for high‐energy photon detection. This configuration enables simultaneous acquisition of high and low energy datasets, which are subsequently processed to generate quantitative parametric maps including RED maps, Z_eff_ maps, and VMIs at various energy levels.

**FIGURE 1:**
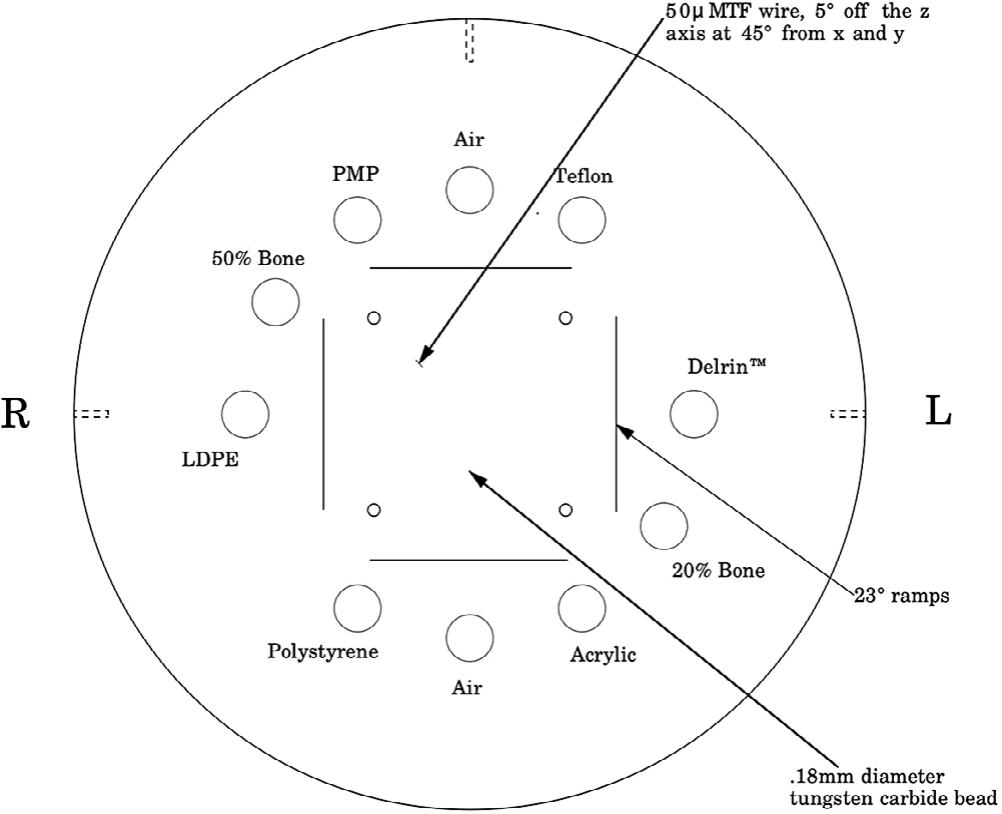
Phantom configuration diagram (image reproduced from the Catphan 604 Product Guide).

This study employed the Catphan 604 phantom^18^ (The Phantom Laboratory, Salem, New York, USA), a standardized calibration device specifically designed for performance evaluation of CT imaging systems. The phantom consists of multiple modular components enclosed within a cylindrical housing with a 20 cm diameter. As shown in Figure 1, the CTP732 module incorporates inserts of well‐characterized reference materials, including air, polytetrafluoroethylene (Teflon), polyoxymethylene (Delrin), 20% bone, polymethyl methacrylate (acrylic), polystyrene, low‐density polyethylene (LDPE), 50% bone, and polymethylpentene (PMP). These materials provide a precisely defined range of physical properties for validating spectral CT quantification. Table 1 summarizes the manufacturer‐specified reference values for each insert's RED values and Z_eff_ values.

**TABLE 1:** Expected values of RED and Z_eff_ for different inserts (reproduced from the Catphan 604 Product Guide).^18^

Material	Expected RED	Expected Z_eff_
Air	0.1	8
Teflon	186.8	8.43
Delin	136.3	6.95
20%Bone	108.4	9.09
Acrylic	114.7	6.47
Polystyrene	99.8	5.70
LDPE	94.5	5.44
50% Bone	131.2	11.46
PMP	85.3	5.44

### 2.2 Image acquisitionjj

All scans were conducted using the Philips Spectral CT 7500 system, with the Catphan 604 phantom positioned at the geometric center of the gantry. A reference protocol served as the baseline for comparative analysis, employing the following parameters: tube voltage of 120 kVp, mAs of 350 mAs, collimation width of 64 × 0.625 mm, pitch of 0.5, field of view (FOV) of 350 mm, slice thickness of 3 mm, UB filter, and iDose 4 iterative reconstruction. To systematically evaluate the impact of acquisition and reconstruction parameters on spectral CT performance, the following variables were independently modified while all other parameters constant: tube voltage of 100, 120, and 140 kVp; mAs of 50, 100, 200, 300, 400, and 500; slice thicknesses of 1, 3, and 5 mm; reconstruction filter including UA, UB, UC, YA, and YB; and pitch of 0.2, 0.3, 0.4, and 0.5. The reconstruction filter includes kernels of different sharpness levels: UA, UB, and UC are head filters (smooth, standard, and sharp, respectively), while YA and YB are sharp and very sharp filters typically used for lung and bone imaging. Table 2 presents the experimental protocol design, including baseline parameters and the systematic variations.

**TABLE 2:** Experimental protocol design for Spectral CT 7500 scanning: Base parameters and variations.

Parameters	Base value	Tested values
Tube voltage	120 kVp	100, 120, 140 kVp
Tube current–time product	350 mAs	50, 100, 200, 300, 400, 500 mAs
Collimation	64*0.625 mm	64*0.625 mm
Pitch	0.5	0.2, 0.3, 0.4, 0.5
Field of view	350 mm	350 mm
Reconstruction filter	UB	UA, UB, UC, YA, YB
Slice thickness	3 mm	1, 3, 5mm
Iterative reconstruction	IDose4	IDose4

For each scan protocol, spectral datasets were reconstructed to generate VMIs at 40, 70, and 100 keV for quantitative comparison with conventional CT (120 kVp single‐energy reconstruction from the dual‐layer system) images. Additionally, material‐specific maps, including Z_eff_ maps and RED maps, were generated using projection‐domain material decomposition algorithms.

### 2.3 Data acquisition and analysis

To minimize partial volume effects and ensure adequate sampling, circular regions of interest (ROIs) with a diameter equal to 75% of the cross‐sectional area of each insert were placed centrally within each insert. These ROIs were then copied to the corresponding locations on other spectral reconstruction images to measure the mean and standard deviation. The measured values included the RED values from the RED maps, the Z_eff_ values from the Z_eff_ maps, and the CT number from VMIs at 40, 70, and 100 keV, as well as from conventional CT images. For RED and Z_eff_, agreement with the manufacturer‐provided reference values was assessed using Bland–Altman analysis. For each scan condition, the mean bias and the 95% limits of agreement (LoA = bias ± 1.96 × SD of the differences) were determined, thereby quantifying both the systematic deviation and the variation in comparison with reference values. To evaluate the stability of CT number, the differences in HU values relative to a reference group (120 kVp, 3 mm, UB reconstruction filter, pitch 0.5, 300 mAs) were calculated. The results are reported as mean ± standard deviation of the relative differences across all scan conditions. All statistical analyses were performed using Python (Version 3.12.5) with NumPy (V2.1.0), SciPy (V1.16.1), and pandas (V2.2.3). Data visualization was generated using Matplotlib (V3.10.0).

## 3 RESULTS

### 3.1 Quantitative accuracy of RED and Z_eff_

Across the full range of tested acquisition and reconstruction parameters, dual‐layer spectral CT demonstrated high quantitative accuracy in the measurement of RED values and Z_eff_ values for various materials inserts.

**FIGURE 2:**
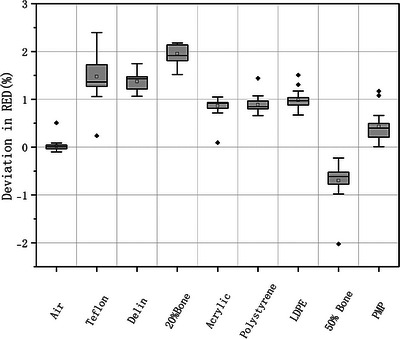
Signed differences between measured and nominal relative electron density (RED) values of inserts across varying acquisition and reconstruction parameters.

**FIGURE 3:**
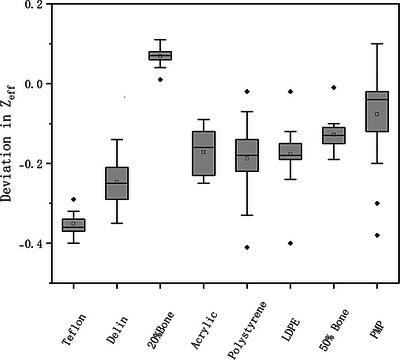
Signed differences between measured and nominal effective atomic number (Z_eff_) values of inserts across varying acquisition and reconstruction parameters.

As shown in Figure 2, the signed differences in measured RED values across various materials inserts remained within 2.4％ of the expected values. Low‐density materials such as polystyrene, LDPE, and PMP exhibited minimal deviation (≤ 1.0％), while high‐density materials, including Teflon and 20% bone, showed larger deviations (≤ 2.4％), though still within acceptable limits.^19^, ^20^ Similarly, Figure 3 presents the signed differences in Z_eff_ measurements values under all acquisition and reconstruction parameters. Most inserts demonstrated the signed differences within 0.4. Notably, polystyrene and PMP showed slightly larger ranges of Z_eff_ deviation compared to other materials. These results confirm that dual‐layer spectral CT can deliver quantitatively accurate RED and Z_eff_ values across a range of material compositions and acquisition and reconstruction parameters.

### 3.2 Dependence of RED and Z_eff_ accuracy on acquisition and reconstruction parameters

**FIGURE 4:**
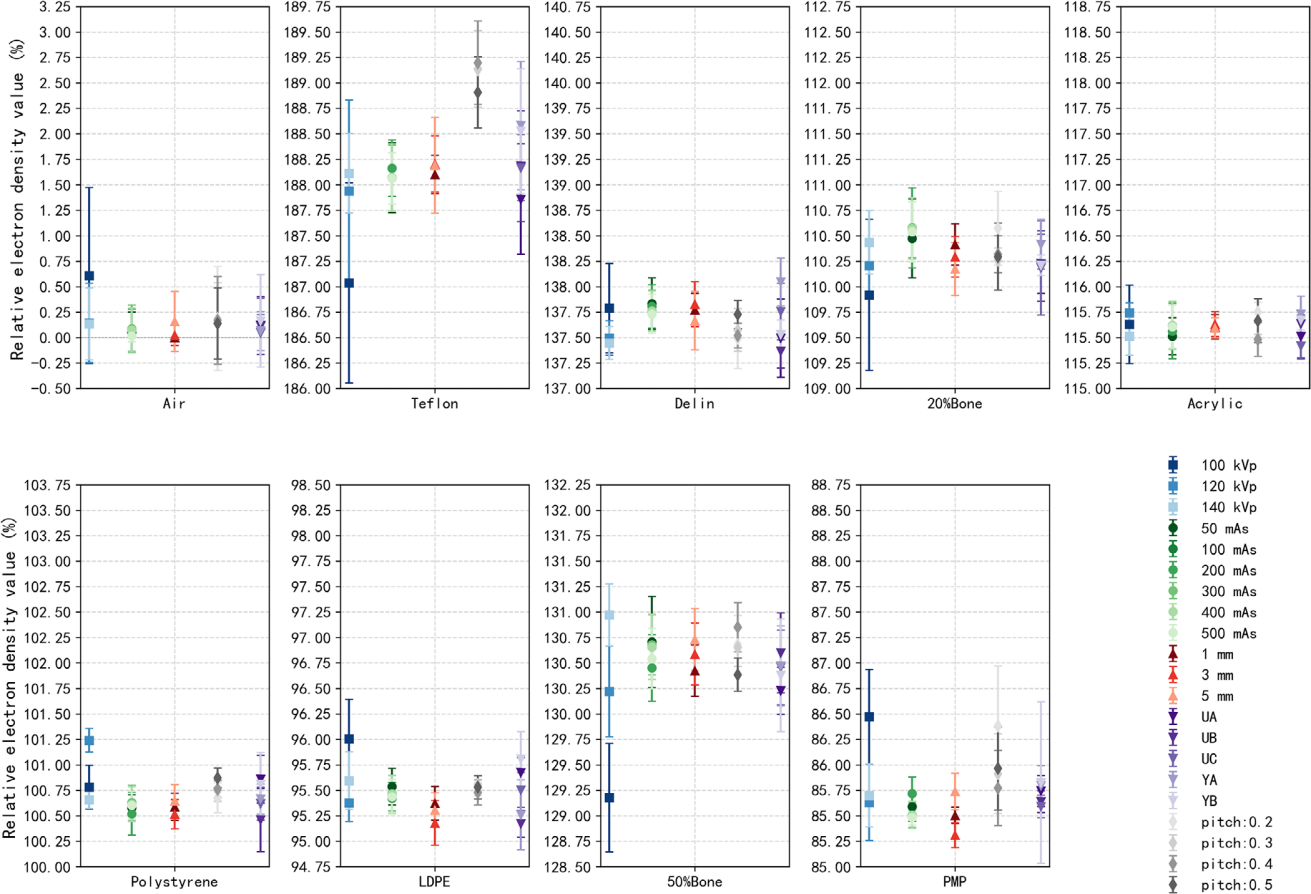
Variation in measured relative electron density (RED) values of each insert under different tube voltages, mAs levels, slice thicknesses, reconstruction filter, and pitch settings.

**FIGURE 5:**
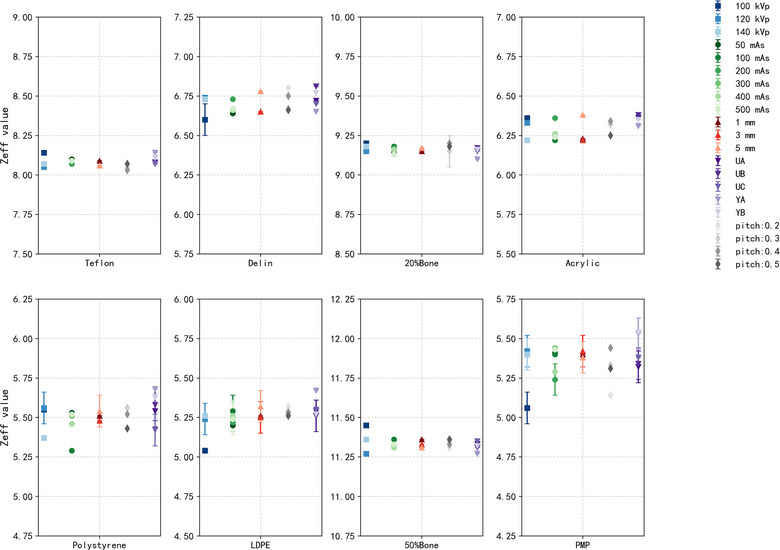
Variation in measured effective atomic number (Z_eff_) values of each insert under different tube voltages, mAs levels, slice thicknesses, reconstruction filter, and pitch settings.

Figures 4 and 5 depict the variation of RED and Z_eff_ values under different acquisition and reconstruction parameters, while Tables S1 and S2 present the Bland–Altman analyses quantifying agreement between the measured and reference values.

For RED, the mean bias across all conditions ranged from 0.70 to 1.03, with 95% LoA generally within ± 3 (Table S1). Figure 4 indicates that tube voltage exhibited the most significant influence, which was confirmed by Bland–Altman analysis: the bias at 100 kVp was 0.70 with LoA from −1.49 to 2.89, compared with narrower intervals at 120 and 140 kVp (−0.89 to 2.42 and −0.52 to 2.18, respectively). Exposure level also affected RED estimates, with 200 mAs showing a slightly higher bias (1.03, LoA −0.71 to 2.78) than higher exposures (300–500 mAs, biases ∼0.93 with similar LoA widths). In contrast, variations in slice thickness, reconstruction filter, and pitch had limited impact, with biases consistently around 0.8 and LoA remaining narrow. Overall, these results demonstrate that tube voltage and exposure level exert more noticeable effects on RED, whereas other parameters show comparatively stable performance, consistent with the distributions observed in Figure 4.

As illustrated in Figure 5, Z_eff_ measurements remained robust across varied acquisition and reconstruction parameters. Among the parameters, tube voltage exerted the most pronounced effect. At 100 kVp, the distribution was broader than at 120 or 140 kVp, which was consistent with Bland–Altman analysis: the bias at 100 kVp was −0.20 with LoA from −0.56 to 0.17, compared with −0.15 (LoA −0.41 to 0.11) and −0.17 (LoA −0.47 to 0.12) at 120 and 140 kVp, respectively (Table S2). In contrast, changes in exposure level, slice thickness (1–5 mm), reconstruction filter, and pitch showed only minor differences, with Figure 5 displaying overlapping distributions and Bland–Altman analysis confirming biases around −0.1 and LoA widths around 0.5 across these conditions.

### 3.3 Dependence of VMI CT number accuracy on acquisition and reconstruction parameters

Figure 6 and Tables S3–S6 summarize the CT‐number differences for VMIs (40, 70, and 100 keV) and for conventional CT under varying acquisition and reconstruction parameters. 40 keV VMI exhibited greater variability, with HU differences up to 27 HU, particularly under low exposure conditions (Table S3). In contrast, the 100 keV VMI showed improved stability, with a maximum HU deviation of only 11 HU (Table S5). The HU distribution of the 70 keV VMI most closely resembled that of conventional CT. The influence of acquisition and reconstruction parameters on VMIs was reduced. Specifically, tube voltage exerted the most pronounced effect on both modalities. For the 70 keV VMI, HU deviations typically remained within 3 HU when tube voltage increased from 100 to 140 kVp (Table S4), whereas in conventional CT the maximum deviation reached 118 HU (Table S6). Slice thickness also influenced the stability of both VMIs and conventional CT, with larger deviations observed at 1 mm reconstructions (Figure 6). Regarding reconstruction filter, UA and UB yielded the most consistent results, with HU deviations generally around 0 ± 3 HU, whereas YA and YB introduced differences of approximately −7 ± 23 HU for high‐density materials such as Teflon at 40 keV. By comparison, variations in exposure level and pitch had smaller effects on both VMIs and conventional CT, with HU deviations generally within 1 ± 3 HU.

FIGURE 6CT number for each insert across VMIs at (a) 40 keV, (b) 70 keV, (c) 100 keV and (d) conventional CT, under varying acquisition and reconstruction parameters.

**Figure:**
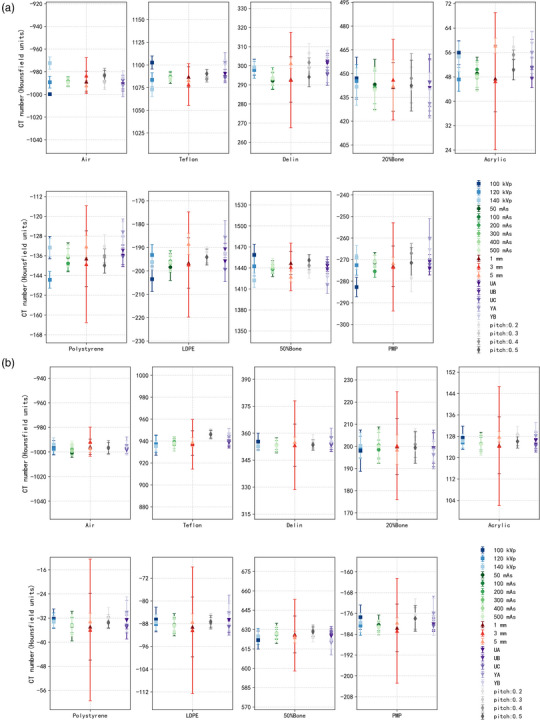

**Figure:**
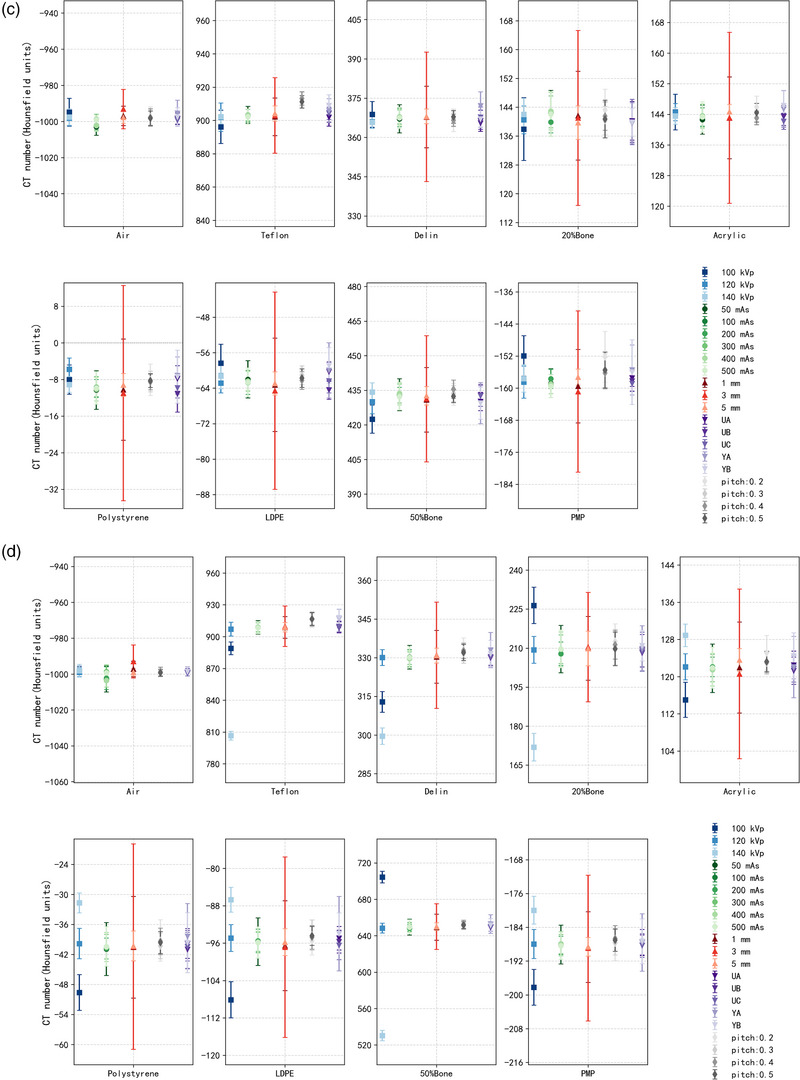

## 4 DISCUSSION

This phantom study systematically evaluated the impact of scan parameters on quantitative accuracy using a second‐generation dual‐layer spectral CT system. The results demonstrated that quantitative measurements, including RED maps, Z_eff_ maps, and VMIs, remained stable across variations in tube voltage, exposure (mAs), reconstruction thickness, pitch, and reconstruction filter. The signed differences in RED remained below 2.4%, those in Z_eff_ were within 0.4, and HU differences of VMIs, especially at 70 keV, were maintained within 8 HU. These findings align with and extend prior reports.^13^ Published data indicate that with a 2% dose error, clinically acceptable tolerance ranges for RED are ± 4.5% in lung, ± 2.2% in fat and muscle, and ± 4.5% in cartilage and cancellous bone, all of which encompass the deviations observed in this study.^19^, ^20^ Similarly, previously reported Z_eff_ deviations of approximately 0.3 are comparable to our results.^14^ With respect to HU reproducibility, tolerances of ± 20 HU for soft tissue and ± 50 HU for lung and bone have been proposed to keep uncertainties in treatment planning dose calculations below 1%.^21^, ^22^ The VMI deviations observed in our analysis fall well within these thresholds, underscoring the robustness of dual‐layer spectral CT for quantitative applications in radiotherapy.

Importantly, tube voltage emerged as the most influential factor across all acquisition and reconstruction parameters. RED measurements at 100 kVp exhibited increased variability compared to 120 or 140 kVp, especially in high‐RED materials such as Teflon and 50% bone. This variability is attributed to the increased x‐ray beam penetration associated with higher kVp values.^23^ While tube voltage caused more variability than other acquisition and reconstruction parameters, all measured values remained within clinically acceptable ranges.^14^, ^19^, ^20^ Dose level also played a role, with low dose showing increased noise and errors. Landry et al.^24^ demonstrated that increasing dose level improved the differentiation of RED and Z_eff_ measurements in tissue‐equivalent inserts scanned with dual‐source DECT. In contrast, Hua et al.^15^ reported that RED and Z_eff_ determinations in dual‐layer DECT remained accurate and stable across the investigated ranges of tube voltage and dose level. This discrepancy may be attributed to technological differences between DECT implementations and the different dose ranges investigated. Hua et al.^15^ focused on relatively higher and narrower dose levels (20–30 mGy), whereas Landry et al.^24^ investigated a considerably wider and generally lower dose range (4.6–18.4 mGy).

Slice thickness and reconstruction filter had a lesser influence on quantitative values. Thinner slices increased statistical noise, as the noise magnitude is approximately inversely proportional to the square root of the slice thickness.^25^, ^26^ The effect of the reconstruction filter was minimal, with UB and UA reconstruction filter producing the most consistent results. Although sharp reconstruction filters are expected to increase image noise due to their emphasis on high‐frequency components, the quantitative impact on RED and Z_eff_ in our study remained minimal. The effect of pitch was limited in our results, as the helical sampling geometry in dual‐layer spectral CT systems has little influence on the accuracy of spectral decomposition. In addition, low‐density and low‐Z materials such as polystyrene and PMP exhibited slightly greater variability, a phenomenon also reported in previous spectral CT phantom studies, which attribute this behavior to reduced spectral separation and higher sensitivity to image noise in such materials^27^.

Analysis of VMIs indicates reduced dependence on tube voltage variations, particularly at 70 keV and 100 keV energy levels. This improvement stems from the monoenergetic reconstruction, which simulates images at a single photon energy and thereby removes the variation in attenuation caused by polychromatic x‐ray spectra.^13^ Comparable results have been observed with twin‐beam spectral CT, where the hardened beam reduces low‐energy photon contributions, leading to lower noise and more consistent VMI CT numbers.^28^ Notably, compared to conventional CT, 70 keV VMIs demonstrated stable performance across different acquisition and reconstruction parameters. These findings corroborate prior clinical reports highlighting VMIs's superior performance in tumor delineation.^29^ Additionally, accurate treatment planning requires high reproducibility of CT number, particularly for dose calculation in treatment planning system (TPS).^30^ Therefore, the measurement reproducibility positions VMIs as particularly valuable for clinical workflows requiring consistent attenuation measurements, including target volume delineation and dose calculation in radiotherapy planning.^31^

This study demonstrates the potential of dual‐layer spectral CT to advance precision radiotherapy. The parameter independence of quantitative measurements with dual‐layer spectral CT enables protocol optimization for specific clinical scenarios. Optimizing CT acquisition and reconstruction parameters can enhance image quality and diagnostic accuracy, particularly for tumor detection and margin delineation.^32^ And optimized scans enables both radiation dose reduction in pediatric imaging and effective metal artifact mitigation, while consistently maintaining accurate material characterization.^4^, ^14^ This capability is particularly valuable for personalized radiotherapy applications.

Despite these encouraging results, several limitations should be acknowledged. First, the study was conducted using a standardized phantom under controlled conditions. While this design ensures reproducibility, it does not fully accommodate the complexities of clinical imaging scenarios involving patient‐specific anatomical variations, motion, and heterogeneous tissue geometries. Second, the analysis was confined to a single dual‐layer spectral CT platform (Philips Spectral CT 7500); results may differ across other spectral CT implementations, such as dual‐source, split‐filter, or rapid kVp‐switching systems. Third, although this study evaluated RED and Z_eff_, future work could include SPR maps to directly assess implications for proton therapy planning.

## 5 CONCLUSION

In summary, this phantom‐based study demonstrates that dual‐layer spectral CT delivers quantitatively accurate RED, Z_eff_, and and VMI CT number across a wide range of acquisition and reconstruction parameters. Compared to conventional CT, VMIs at 70 keV provided consistent CT number with minimal dependence on acquisition and reconstruction parameters. These findings contribute valuable insights toward the standardization and optimization of spectral CT protocols in clinical radiation oncology, with potential to enhance critical radiotherapy workflows including target delineation and dose calculation accuracy.

## AUTHOR CONTRIBUTIONS

*Conception and design*: Lingxuan Leng, Qizhen Zhu, Bo Yang, and Jie Qiu. *Administrative support*: Jie Qiu and Bo Yang. *Provision of study materials or patients*: HuiYing Qu and Bing Zhou. *Collection and assembly of data*: Lingxuan Leng, Qizhen Zhu, Wenbo Li, and Fuquan Zhang. *Data analysis and interpretation*: Lingxuan Leng and Qizhen Zhu. *Manuscript writing*: All authors. *Final approval of manuscript*: All authors.

## CONFLICT OF INTEREST STATEMENT

All authors have completed the ICMJE uniform disclosure form. The authors have no conflicts of interest to declare.

## Supporting information



Supporting Information
