# Supplementary material for: Impact of acquisition and reconstruction parameters on quantitative accuracy in dual‐layer spectral CT: A phantom study
Source: J Appl Clin Med Phys. 2025 Dec 15;26(12):e70423. doi: 10.1002/acm2.70423 (PMC12703825; doi:10.1002/acm2.70423)
Supplement: Supplementary file 1 — Supporting Information [file ACM2-26-e70423-s001.docx]

Table 1. Bland–Altman analysis of RED (bias and 95% LoA) under different acquisition and reconstruction parameters

| Condition | Bias | SD_diff | LoA_lower | LoA_upper |
| --- | --- | --- | --- | --- |
| 100kVp | 0.70 | 1.12 | -1.49 | 2.89 |
| 120kVp | 0.77 | 0.84 | -0.89 | 2.42 |
| 140kVp | 0.83 | 0.69 | -0.52 | 2.18 |
| 1mm | 0.81 | 0.80 | -0.76 | 2.37 |
| 3mm | 0.80 | 0.82 | -0.81 | 2.42 |
| 5mm | 0.80 | 0.86 | -0.89 | 2.49 |
| UA | 0.81 | 0.82 | -0.79 | 2.42 |
| UB | 0.80 | 0.82 | -0.81 | 2.42 |
| UC | 0.77 | 0.84 | -0.89 | 2.42 |
| YA | 0.74 | 0.85 | -0.93 | 2.41 |
| YB | 0.72 | 0.83 | -0.90 | 2.34 |
| pitch0.2 | 0.79 | 0.70 | -0.58 | 2.16 |
| pitch0.3 | 0.73 | 0.81 | -0.86 | 2.32 |
| pitch0.4 | 0.70 | 0.73 | -0.73 | 2.12 |
| pitch0.5 | 0.74 | 0.80 | -0.83 | 2.30 |
| 50mAs | 0.88 | 0.90 | -0.88 | 2.64 |
| 100mAs | 0.87 | 0.84 | -0.77 | 2.51 |
| 200mAs | 1.03 | 0.89 | -0.71 | 2.78 |
| 300mAs | 0.93 | 0.87 | -0.78 | 2.64 |
| 400mAs | 0.94 | 0.85 | -0.73 | 2.61 |
| 500mAs | 0.93 | 0.90 | -0.84 | 2.70 |

Bias is the mean difference between measured and reference values; LoA, limits of agreement = bias ± 1.96 × SD of the differences.

Relative electron density, RED

Table 2. Bland–Altman analysis of Z_eff_ (bias and 95% LoA) under different acquisition and reconstruction parameters

| Condition | Bias | SD_diff | LoA_lower | LoA_upper |
| --- | --- | --- | --- | --- |
| 100kVp | -0.20 | 0.19 | -0.56 | 0.17 |
| 120kVp | -0.15 | 0.13 | -0.41 | 0.11 |
| 140kVp | -0.17 | 0.15 | -0.47 | 0.12 |
| 1mm | -0.17 | 0.13 | -0.43 | 0.09 |
| 3mm | -0.18 | 0.17 | -0.50 | 0.14 |
| 5mm | -0.17 | 0.12 | -0.41 | 0.07 |
| UA | -0.17 | 0.13 | -0.42 | 0.09 |
| UB | -0.19 | 0.12 | -0.43 | 0.06 |
| UC | -0.17 | 0.13 | -0.42 | 0.09 |
| YA | -0.17 | 0.13 | -0.43 | 0.10 |
| YB | -0.18 | 0.15 | -0.46 | 0.11 |
| pitch0.2 | -0.13 | 0.13 | -0.38 | 0.12 |
| pitch0.3 | -0.15 | 0.12 | -0.38 | 0.08 |
| pitch0.4 | -0.13 | 0.12 | -0.36 | 0.10 |
| pitch0.5 | -0.16 | 0.13 | -0.42 | 0.10 |
| 50mAs | -0.11 | 0.15 | -0.39 | 0.17 |
| 100mAs | -0.10 | 0.14 | -0.37 | 0.16 |
| 200mAs | -0.16 | 0.13 | -0.42 | 0.09 |
| 300mAs | -0.16 | 0.13 | -0.42 | 0.10 |
| 400mAs | -0.14 | 0.15 | -0.43 | 0.16 |
| 500mAs | -0.18 | 0.14 | -0.46 | 0.09 |

Effective atomic number, Z_eff_

Table 3. HU differences (mean±SD) of 40 keV VMI relative to reference group under different acquisition and reconstruction parameters.

| Material/  Parameter | Air | Teflon | Delin | 20%Bone | Acrylic | Polystyrene | LDPE | 50%Bone | PMP |
| --- | --- | --- | --- | --- | --- | --- | --- | --- | --- |
| 100kVp | -11 ± 1 | 19 ± 7 | 1 ± 4 | 2 ± 13 | 9 ± 4 | 13 ± 5 | -10 ± 5 | 16 ± 15 | -10 ± 4 |
| 120kVp | 0 ± 5 | 0 ± 8 | 0 ± 4 | 0 ± 10 | 0 ± 4 | 0 ± 3 | 0 ± 4 | 0 ± 14 | 0 ± 5 |
| 140kVp | 17 ± 6 | -11 ± 8 | 1 ± 4 | -4 ± 12 | 7 ± 5 | 13 ± 4 | -3 ± 3 | -20 ± 10 | 4 ± 6 |
| 1mm | 0 ± 4 | 0 ± 6 | -2 ± 5 | 3 ± 16 | 1 ± 5 | -1 ± 6 | -2 ± 6 | -1 ± 11 | 0 ± 5 |
| 3mm | 0 ± 4 | 0 ± 4 | 0 ± 3 | 0 ± 14 | 0 ± 4 | 0 ± 4 | 0 ± 3 | 0 ± 7 | 0 ± 3 |
| 5mm | 1 ± 4 | 1 ± 6 | 0 ± 6 | 1 ± 10 | 3 ± 3 | -3 ± 3 | 1 ± 5 | -2 ± 6 | -4 ± 3 |
| UA | 1 ± 4 | 0 ± 4 | -1 ± 3 | 0 ± 13 | 1 ± 4 | 0 ± 3 | 0 ± 3 | 0 ± 5 | 0 ± 3 |
| UB | 0 ± 4 | 0 ± 4 | 0 ± 3 | 0 ± 14 | 0 ± 4 | 0 ± 4 | 0 ± 3 | 0 ± 7 | 0 ± 3 |
| UC | 0 ± 4 | 0 ± 5 | 0 ± 4 | -1 ± 13 | 0 ± 5 | 0 ± 5 | 0 ± 4 | 2 ± 11 | 0 ± 3 |
| YA | 1 ± 9 | 1 ± 11 | -1 ± 12 | 1 ± 15 | 0 ± 11 | -1 ± 11 | 0 ± 11 | 7 ± 16 | -2 ± 9 |
| YB | 6 ± 16 | -7 ± 23 | -1 ± 25 | 6 ± 25 | -1 ± 22 | -3 ± 24 | -1 ± 23 | 1 ± 34 | -2 ± 20 |
| pitch0.2 | -4 ± 4 | -3 ± 3 | 6 ± 3 | -2 ± 16 | 7 ± 2 | 4 ± 4 | 11 ± 4 | -19 ± 8 | 1 ± 3 |
| pitch0.3 | -4 ± 5 | 4 ± 5 | 6 ± 5 | 0 ± 15 | 5 ± 4 | 1 ± 4 | 4 ± 3 | -3 ± 9 | -2 ± 3 |
| pitch0.4 | -3 ± 4 | 1 ± 5 | 7 ± 3 | -3 ± 18 | -3 ± 3 | 3 ± 6 | 9 ± 2 | -8 ± 7 | 2 ± 3 |
| pitch0.5 | 0 ± 5 | 0 ± 5 | 0 ± 6 | 0 ± 18 | 0 ± 3 | 0 ± 4 | 0 ± 5 | 0 ± 9 | 0 ± 3 |
| 50mAs | -3 ± 9 | 16 ± 11 | -2 ± 6 | -10 ± 9 | 1 ± 7 | 9 ± 5 | 8 ± 7 | -27 ± 12 | 11 ± 9 |
| 100mAs | 3 ± 7 | 13 ± 6 | 5 ± 4 | -2 ± 11 | 4 ± 4 | 8 ± 6 | 1 ± 4 | -16 ± 12 | 6 ± 5 |
| 200mAs | 0 ± 5 | 0 ± 6 | 8 ± 5 | 0 ± 13 | 3 ± 3 | 4 ± 5 | 2 ± 2 | -9 ± 8 | -7 ± 6 |
| 300mAs | 0 ± 7 | 0 ± 3 | 0 ± 3 | 0 ± 13 | 0 ± 2 | 0 ± 3 | 0 ± 3 | 0 ± 8 | 0 ± 6 |
| 400mAs | 7 ± 6 | -3 ± 3 | 3 ± 2 | 6 ± 16 | 0 ± 2 | 0 ± 3 | 0 ± 2 | 2 ± 6 | 4 ± 5 |
| 500mAs | 6 ± 5 | 3 ± 5 | -5 ± 5 | 1 ± 16 | -5 ± 3 | -4 ± 3 | 0 ± 3 | 9 ± 8 | 0 ± 7 |

Reference group in each subgroup was defined as 120 kVp, 3 mm, UB, pitch 0.5 and 300 mAs.

Table 4. HU differences (mean±SD) of 70 keV VMI relative to reference group under different acquisition and reconstruction parameters.

| Material/  Parameter | Air | Teflon | Delin | 20%Bone | Acrylic | Polystyrene | LDPE | 50%Bone | PMP |
| --- | --- | --- | --- | --- | --- | --- | --- | --- | --- |
| 100kVp | 1 ± 6 | -1 ± 9 | 3 ± 5 | -2 ± 9 | 2 ± 4 | 1 ± 3 | 2 ± 4 | -3 ± 7 | 3 ± 5 |
| 120kVp | 0 ± 4 | 0 ± 8 | 0 ± 2 | 0 ± 5 | 0 ± 2 | 0 ± 3 | 0 ± 2 | 0 ± 6 | 0 ± 4 |
| 140kVp | 3 ± 5 | -2 ± 4 | 0 ± 3 | 1 ± 6 | 1 ± 3 | 0 ± 2 | 1 ± 3 | 0 ± 5 | 1 ± 4 |
| 1mm | -1 ± 4 | 1 ± 5 | 0 ± 4 | 1 ± 8 | 0 ± 4 | 0 ± 5 | 0 ± 4 | 0 ± 8 | 1 ± 4 |
| 3mm | 0 ± 4 | 0 ± 7 | 0 ± 3 | 0 ± 8 | 0 ± 3 | 0 ± 3 | 0 ± 2 | 0 ± 5 | 0 ± 2 |
| 5mm | 0 ± 3 | 2 ± 3 | 0 ± 4 | -1 ± 4 | 0 ± 2 | 0 ± 2 | 0 ± 3 | -1 ± 4 | 0 ± 2 |
| UA | 1 ± 3 | 1 ± 3 | 0 ± 2 | 1 ± 6 | 0 ± 3 | 0 ± 2 | 0 ± 2 | 0 ± 4 | 0 ± 2 |
| UB | 0 ± 4 | 0 ± 7 | 0 ± 3 | 0 ± 8 | 0 ± 3 | 0 ± 3 | 0 ± 2 | 0 ± 5 | 0 ± 2 |
| UC | 5 ± 3 | 1 ± 4 | 0 ± 4 | 0 ± 7 | 0 ± 4 | 0 ± 4 | 0 ± 3 | 0 ± 6 | 0 ± 3 |
| YA | 3 ± 6 | 1 ± 11 | 0 ± 12 | 0 ± 13 | 0 ± 11 | 0 ± 11 | -1 ± 11 | -1 ± 14 | 0 ± 9 |
| YB | 8 ± 12 | 0 ± 23 | 0 ± 25 | 0 ± 24 | -1 ± 22 | -1 ± 23 | -2 ± 22 | -1 ± 28 | -2 ± 20 |
| pitch0.2 | 2 ± 3 | -1 ± 4 | 1 ± 3 | -1 ± 7 | 3 ± 2 | 1 ± 3 | 2 ± 3 | -4 ± 4 | 1 ± 2 |
| pitch0.3 | 1 ± 3 | -1 ± 5 | 0 ± 4 | 0 ± 7 | 2 ± 3 | 2 ± 3 | 2 ± 2 | -3 ± 5 | 1 ± 2 |
| pitch0.4 | 1 ± 4 | 0 ± 5 | -1 ± 2 | -1 ± 8 | 1 ± 2 | -1 ± 4 | 0 ± 2 | -2 ± 5 | 1 ± 3 |
| pitch0.5 | 0 ± 3 | 0 ± 4 | 0 ± 4 | 0 ± 8 | 0 ± 2 | 0 ± 2 | 0 ± 3 | 0 ± 5 | 0 ± 2 |
| 50mAs | 2 ± 7 | -3 ± 7 | 4 ± 5 | -3 ± 6 | 2 ± 5 | 3 ± 4 | 3 ± 6 | -8 ± 9 | 2 ± 7 |
| 100mAs | 1 ± 6 | -1 ± 6 | 1 ± 3 | -1 ± 5 | 3 ± 4 | 2 ± 6 | 2 ± 4 | -6 ± 6 | 1 ± 7 |
| 200mAs | 1 ± 5 | 0 ± 5 | 1 ± 4 | 2 ± 7 | 3 ± 3 | 1 ± 4 | 0 ± 2 | -2 ± 5 | 2 ± 6 |
| 300mAs | 0 ± 5 | 0 ± 4 | 0 ± 3 | 0 ± 5 | 0 ± 2 | 0 ± 2 | 0 ± 2 | 0 ± 4 | 0 ± 5 |
| 400mAs | 1 ± 6 | 0 ± 3 | 0 ± 2 | 2 ± 6 | 0 ± 2 | 0 ± 2 | -1 ± 2 | 2 ± 4 | 0 ± 4 |
| 500mAs | 0 ± 5 | -1 ± 4 | 0 ± 3 | 1 ± 7 | 0 ± 3 | 0 ± 2 | -1 ± 2 | 1 ± 4 | 0 ± 5 |

Reference group in each subgroup was defined as 120 kVp, 3 mm, UB, pitch 0.5 and 300 mAs.

Table 5. HU differences (mean±SD) of 100 keV VMI relative to reference group under different acquisition and reconstruction parameters.

| Material/  Parameter | Air | Teflon | Delin | 20%Bone | Acrylic | Polystyrene | LDPE | 50%Bone | PMP |
| --- | --- | --- | --- | --- | --- | --- | --- | --- | --- |
| 100kVp | 3 ± 8 | -6 ± 10 | 3 ± 5 | -3 ± 9 | 0 ± 5 | -2 ± 3 | 4 ± 4 | -8 ± 6 | 7 ± 5 |
| 120kVp | 0 ± 4 | 0 ± 8 | 0 ± 2 | 0 ± 4 | 0 ± 2 | 0 ± 2 | 0 ± 2 | 0 ± 5 | 0 ± 4 |
| 140kVp | 1 ± 4 | 0 ± 4 | 0 ± 2 | 2 ± 4 | -1 ± 2 | -3 ± 2 | 2 ± 3 | 4 ± 4 | 1 ± 3 |
| 1mm | -1 ± 5 | 0 ± 5 | -1 ± 5 | -1 ± 6 | -1 ± 4 | 0 ± 4 | 0 ± 4 | 0 ± 7 | 0 ± 4 |
| 3mm | 0 ± 3 | 0 ± 4 | 0 ± 3 | 0 ± 5 | 0 ± 3 | 0 ± 3 | 0 ± 2 | 0 ± 5 | 0 ± 2 |
| 5mm | 0 ± 2 | 0 ± 4 | 0 ± 4 | -3 ± 3 | 0 ± 2 | 0 ± 2 | 0 ± 3 | -1 ± 3 | 1 ± 2 |
| UA | 3 ± 3 | 0 ± 3 | 0 ± 3 | 0 ± 4 | 0 ± 3 | 0 ± 2 | 0 ± 2 | 0 ± 4 | 0 ± 2 |
| UB | 0 ± 3 | 0 ± 4 | 0 ± 3 | 0 ± 5 | 0 ± 3 | 0 ± 3 | 0 ± 2 | 0 ± 5 | 0 ± 2 |
| UC | 3 ± 2 | -1 ± 5 | 0 ± 4 | -1 ± 6 | 0 ± 4 | 0 ± 4 | 0 ± 3 | -1 ± 6 | -1 ± 3 |
| YA | 5 ± 5 | -1 ± 11 | 0 ± 12 | -1 ± 12 | 0 ± 11 | 0 ± 11 | -1 ± 11 | -3 ± 14 | -1 ± 9 |
| YB | 9 ± 11 | 0 ± 23 | 0 ± 25 | -2 ± 24 | 0 ± 22 | -1 ± 23 | -2 ± 22 | -2 ± 27 | -2 ± 20 |
| pitch0.2 | 2 ± 3 | 0 ± 5 | 0 ± 3 | 0 ± 5 | 3 ± 2 | 1 ± 2 | -1 ± 3 | 0 ± 4 | 2 ± 2 |
| pitch0.3 | 1 ± 3 | -2 ± 5 | -1 ± 4 | 0 ± 5 | 1 ± 3 | 2 ± 3 | 2 ± 2 | -2 ± 4 | 1 ± 2 |
| pitch0.4 | 1 ± 3 | 0 ± 5 | -2 ± 2 | 0 ± 6 | 1 ± 2 | -1 ± 4 | -2 ± 2 | 0 ± 5 | 1 ± 3 |
| pitch0.5 | 0 ± 2 | 0 ± 4 | 0 ± 4 | 0 ± 6 | 0 ± 2 | 0 ± 2 | 0 ± 3 | 0 ± 5 | 0 ± 2 |
| 50mAs | 3 ± 7 | -7 ± 7 | 5 ± 5 | -1 ± 6 | 2 ± 5 | 1 ± 4 | 1 ± 6 | -4 ± 9 | 0 ± 6 |
| 100mAs | 0 ± 5 | -4 ± 6 | 0 ± 3 | 0 ± 4 | 3 ± 4 | 1 ± 6 | 2 ± 4 | -4 ± 7 | 0 ± 8 |
| 200mAs | 1 ± 5 | 0 ± 5 | -1 ± 4 | 3 ± 6 | 3 ± 3 | 0 ± 3 | -1 ± 2 | 0 ± 5 | 4 ± 6 |
| 300mAs | 0 ± 5 | 0 ± 4 | 0 ± 3 | 0 ± 4 | 0 ± 2 | 0 ± 2 | 0 ± 2 | 0 ± 3 | 0 ± 4 |
| 400mAs | 0 ± 5 | 0 ± 4 | -1 ± 2 | 1 ± 4 | 0 ± 2 | 0 ± 2 | -1 ± 2 | 2 ± 4 | 0 ± 4 |
| 500mAs | 0 ± 4 | -2 ± 4 | 1 ± 2 | 0 ± 5 | 1 ± 2 | 0 ± 2 | -1 ± 2 | -1 ± 3 | 0 ± 4 |

Reference group in each subgroup was defined as 120 kVp, 3 mm, UB, pitch 0.5 and 300 mAs.

Table 6. HU differences (mean±SD) of conventional CT relative to reference group under different acquisition and reconstruction parameters.

| Material/  Parameter | Air | Teflon | Delin | 20%Bone | Acrylic | Polystyrene | LDPE | 50%Bone | PMP |
| --- | --- | --- | --- | --- | --- | --- | --- | --- | --- |
| 100kVp | 0 ± 3 | -18 ± 6 | -17 ± 4 | 17 ± 7 | -7 ± 4 | -10 ± 4 | -13 ± 4 | 56 ± 6 | -10 ± 4 |
| 120kVp | 0 ± 3 | 0 ± 7 | 0 ± 3 | 0 ± 5 | 0 ± 3 | 0 ± 3 | 0 ± 3 | 0 ± 5 | 0 ± 3 |
| 140kVp | 1 ± 4 | -101 ± 4 | -31 ± 3 | -37 ± 5 | 7 ± 2 | 8 ± 2 | 8 ± 3 | -118 ± 6 | 8 ± 3 |
| 1mm | 1 ± 7 | 0 ± 6 | 0 ± 5 | 0 ± 9 | 0 ± 5 | 0 ± 5 | 0 ± 5 | 0 ± 9 | 0 ± 5 |
| 3mm | 0 ± 4 | 0 ± 5 | 0 ± 3 | 0 ± 7 | 0 ± 4 | 0 ± 3 | 0 ± 2 | 0 ± 5 | 0 ± 3 |
| 5mm | 1 ± 6 | 0 ± 4 | 0 ± 3 | -2 ± 4 | 1 ± 2 | 0 ± 2 | 0 ± 2 | -1 ± 4 | 0 ± 2 |
| UA | 5 ± 4 | 0 ± 4 | 0 ± 2 | 0 ± 6 | 0 ± 3 | 0 ± 2 | 0 ± 2 | 0 ± 4 | 0 ± 2 |
| UB | 0 ± 4 | 0 ± 5 | 0 ± 3 | 0 ± 7 | 0 ± 4 | 0 ± 3 | 0 ± 2 | 0 ± 5 | 0 ± 3 |
| UC | 4 ± 2 | 0 ± 6 | 0 ± 4 | 0 ± 7 | 0 ± 4 | 0 ± 4 | 0 ± 3 | 0 ± 8 | 0 ± 3 |
| YA | 6 ± 4 | 0 ± 10 | 1 ± 10 | 0 ± 12 | 0 ± 10 | 0 ± 10 | -1 ± 10 | 0 ± 14 | -1 ± 8 |
| YB | 11 ± 9 | 1 ± 19 | 1 ± 21 | 1 ± 21 | -1 ± 18 | 0 ± 21 | -1 ± 19 | 1 ± 25 | -1 ± 17 |
| pitch0.2 | 1 ± 2 | -1 ± 4 | 0 ± 3 | 1 ± 7 | 2 ± 2 | 0 ± 3 | 1 ± 3 | 0 ± 4 | 0 ± 2 |
| pitch0.3 | 0 ± 2 | -1 ± 5 | 0 ± 4 | 1 ± 7 | 1 ± 3 | 1 ± 3 | 2 ± 3 | 0 ± 5 | 0 ± 2 |
| pitch0.4 | 1 ± 2 | 0 ± 5 | 0 ± 3 | -1 ± 7 | 0 ± 3 | -1 ± 4 | 1 ± 2 | -1 ± 4 | 1 ± 3 |
| pitch0.5 | 0 ± 1 | 0 ± 5 | 0 ± 3 | 0 ± 7 | 0 ± 3 | 0 ± 3 | 0 ± 3 | 0 ± 5 | 0 ± 3 |
| 50mAs | 1 ± 3 | 0 ± 9 | 1 ± 7 | 0 ± 9 | -1 ± 7 | 2 ± 7 | 0 ± 8 | 0 ± 10 | 0 ± 7 |
| 100mAs | 1 ± 3 | 1 ± 8 | 0 ± 5 | 1 ± 6 | 2 ± 5 | 1 ± 6 | 0 ± 4 | -1 ± 7 | 0 ± 5 |
| 200mAs | 0 ± 2 | 0 ± 8 | 2 ± 5 | 3 ± 7 | 2 ± 4 | 1 ± 4 | -1 ± 3 | -1 ± 5 | 0 ± 4 |
| 300mAs | 0 ± 2 | 0 ± 6 | 0 ± 4 | 0 ± 6 | 0 ± 2 | 0 ± 3 | 0 ± 3 | 0 ± 4 | 0 ± 3 |
| 400mAs | 0 ± 3 | -1 ± 6 | 1 ± 3 | 1 ± 6 | 0 ± 2 | 1 ± 3 | 0 ± 3 | 0 ± 4 | 1 ± 3 |
| 500mAs | 0 ± 2 | 0 ± 6 | 0 ± 3 | 0 ± 6 | 0 ± 2 | 1 ± 2 | 0 ± 2 | -1 ± 4 | 1 ± 3 |

Reference group in each subgroup was defined as 120 kVp, 3 mm, UB, pitch 0.5 and 300 mAs.
